# Aggregated Nanotransfersomal Dry Powder Inhalation of Itraconazole for Pulmonary Drug Delivery

**DOI:** 10.15171/apb.2016.009

**Published:** 2016-03-17

**Authors:** Mehdi Hassanpour Aghdam, Saeed Ghanbarzadeh, Yousef Javadzadeh, Hamed Hamishehkar

**Affiliations:** ^1^ Research Center for Pharmaceutical Nanotechnology, and Students’ Research Committee, Tabriz University of Medical Sciences, Tabriz, Iran.; ^2^ Zanjan Pharmaceutical Nanotechnology Research Center, and Department of Pharmaceutics, Faculty of Pharmacy, Zanjan University of Medical Sciences, Zanjan, Iran.; ^3^ Drug Applied Research Center, Tabriz University of Medical Sciences, Tabriz, Iran.; ^4^ Biotechnology Research Center and Department of Pharmaceutics, Faculty of Pharmacy, Tabriz University of Medical Sciences, Tabriz, Iran.

**Keywords:** Pulmonary drug delivery, Dry powder inhaler, Transfersome, Itraconazole, DPI

## Abstract

***Purpose:*** Local therapy is a valuable and strategic approach in the treatment of lung associated diseases and dry powder inhalation (DPI) formulations play the key role in this plan. Transfersome has been introduced as a novel biocompatible vesicular system with potential for administration in pulmonary drug delivery. The present study was designed to prepare Itraconazole-loaded nanotrantransfersomal DPI formulation.

***Methods:*** Itraconazole-loaded nanotransfersomes with three different types of surfactant in varying concentrations were prepared and characterized in the point of particle size distribution and morphology by laser light scattering and scanning electron microscopy (SEM) methods. The optimized transferosomal formulations were co-spray dried with mannitol and the aerosolization efficiency and aerodynamic properties of dry powders were determined by next generation impactor using a validated HPLC technique.

***Results:*** The volume mean diameter of optimized nanotransfersomal formulation with lecithin:Span® 60 in the ratio of 90:10 was 171 nm with narrow size distribution pattern which increased up to 518 nm after drug loading. Different types of surfactant did not influence the particle size significantly. SEM images confirmed the formation of aggregated nanoparticles in the suitable range (1-5 µm) for the pulmonary drug delivery. Aerosolization evaluation of co-spray dried formulations with different amounts of mannitol indicated that 2:1 ratio of mannitol:transfersome (w:w) showed the best aerosolization efficiency (fine particle fraction (FPF)=37%). Increasing of mannitol significantly decreased the FPF of the optimized formulations.

***Conclusion:*** The results of this study was introduced the potential application of nanotransfersomes in the formulation of DPIs for lung delivery of various drugs.

## Introduction

Itraconazole is a broad-spectrum synthetic triazole antifungal agent which is active against a broad spectrum of fungal species including *Cryptococcus, Candida, Aspergillus, Blastomyces* and *Histoplasma capsulatum.*^1^ The lungs are suitable for both local and systemic drug delivery due to the presence of a large alveolar surface area with high permeability owing to their thin epithelial layer.^2^ Development of drug delivery systems for pulmonary application had received considerable attention and high patient compliance in the last three decades due to be a noninvasive method of drug administration. Furthermore, the possibility to locally and more site-specific drug delivery at high concentrations into the diseased lung by avoiding the first-pass metabolism and reduced systemic doses would lead to maximum therapeutic efficiency and minimum side effects, respectively.^3-6^ The use of liposomes as drug carriers for pulmonary delivery has been reported for different kinds of therapeutics.^7,8^ The utilization of lipidic vesicular systems for pulmonary drug delivery has many potential advantages over aerosol delivery of the corresponding non-encapsulated drug, including carrier suitability for most lipophilic drugs, compatibility and reducing local irritation of lung tissue, prolonging local and systemic therapeutic drug levels and finally facilitating intracellular drug delivery especially to alveolar macrophages, tumor cells or epithelial cells.^9^ Intensive research over the past 25 years led to the introduction and development of a new class of highly deformable liposomes resembling the natural cell vesicle, named transferosomes.^10-12^ Transfersomes are composed of phospholipids like phosphatidylcholine and edge activators which self assembles into lipid bilayer in aqueous environment and closes to form a vesicle.^13,14^ An edge activator is often a single-chain surfactant with a high radius of curvature that destabilizes the lipid bilayers of the vesicles and increases the deformability of the bilayers.^15^ Spray drying has been considered as a simple one-step process for producing small particles for pulmonary administration, that can be easily scaled up.^16-18^ Pressurized metered-dose inhalers (MDI), nebulizers, and dry powder inhalers (DPI) are the three main delivery systems used for aerosol inhalation in humans. Among these, DPI appears to be the most promising for future use. They are propellant-free, portable, easy to operate and low-cost devices with improved stability of the formulation as a result of the dry state.^19,20^ According to our literature review, there is no report about the application of nanotransfersomes in pulmonary drug delivery. Therefore, the primary aim of this study was to introduce transfersomes as a carrier for pulmonary delivery in the form of dry powder inhalation formulation.

## Materials and Methods

### Materials

Acetonitrile, Tween^®^80, Ursodiol, Chloroform and Ortho-phosphoric acid were purchased from Merck Chemicals Co. (Germany). Soybean phosphatidylcholine (SPC, purity >99%) was purchased from Lipoid GmbH (Ludwigshafen, Germany). Itraconazole was supplied from Cipla Company (India). Span^®^60 and Span^®^80 were supplied from Sigma Company (USA) and BDH laboratory (UK), respectively. D-Mannitol and ethyl alcohol were obtained from Fluka (Germany) and JATA (Iran) Companies, respectively.

### Methods

#### Preparation of nanotransfersomes

Transfersomes were prepared by the thin film hydration method. Briefly, desired amounts of SPC (100 mg), Itraconazole (5 mg) and surfactant (5, 10, and 15 mg) were dissolved in an appropriate volume of organic solvent (Table 1). The container tightly closed, protected from light and maintained at room temperature for one day to make sure of formation of complete and homogeneous solution. The mixture was transformed to a round-bottomed flask for solvent removal using a rotary evaporator (Heidolph, Germany) at reduced pressure and above phase transition temperature of lecithin (60 °C). The dried film was hydrated for one hour at 60 °C and then sonicated using a prob sonicator (Sonix, Vibracell), with 0.5 sec on and 0.5 sec off intervals, for a total period of 15 min.^12,21^

**Table 1:** Composition of various nanotransfersome formulations. Data was presented as mean ± standard deviation (n=3).

**Formulation code**	**Type of Surfactant**	**Surfactant (mg)**	**Itraconazole (mg)**	**VMD** ^a^ **(nm)**	**Span**
**T1**	Span^®^80	10	0	360 ± 21	1.18 ± 0.03
**T2**	Span^®^80	5	5	556 ± 29	1.44 ± 0.04
**T3**	Span^®^80	10	5	526 ± 31	1.74 ± 0.03
**T4**	Span^®^80	15	5	598 ± 34	1.70 ± 0.06
**T5**	Span^®^60	10	0	171 ± 28	1.03 ± 0.03
**T6**	Span^®^60	5	5	535 ± 22	1.33 ± 0.03
**T7**	Span^®^60	10	5	518 ± 23	1.07 ± 0.03
**T8**	Span^®^60	15	5	534 ± 30	2.16 ± 0.14
**T9**	Ursodiol	5	5	545 ± 22	1.17 ± 0.04
**T10**	Ursodiol	10	5	522 ± 27	1.04 ± 0.04
**T11**	Ursodiol	15	5	594 ± 41	1.66 ± 0.04

^a^ Volume median diameter

#### Spray drying process

For producing DPI formulations, different amounts of mannitol powder (100, 200, 300 and 600 mg named as F1, F2, F3 and F4, respectively) was co-spray dried with the nanotransfersomal dispersion (100 mg) with desired characteristics using a mini spray dryer at an inlet temperature of 100 ± 5 °C, outlet temperature of 60 ± 5 °C, aspiration setting of 90% and spray flow of 400 NL/h. Immediately after powder collection from the cyclone of spray dryer, spray-dried particles were packed into the tightly closed container wrapped in aluminum foil and desiccated over silica gel at room temperature until further studies.

#### Particle size analysis

The particle size and particle size distribution of prepared nanotransfersomal dispersions were determined by particle size analyzer (Wing SALD 2101, Japan). The size distribution was expressed by the volume median diameter (VMD) and span value. The smaller span values correspond to narrower size distribution. Span value calculated using following equation:

$$\text{Span=}\frac{\text{D}\left(\text{v90\%}\right)\text{-D}\left(\text{v10\%}\right)}{\text{D}\left(\text{v50\%}\right)}$$

Where D(v,90), D(v,10) and D(v,50) are the equivalent volume diameters at 90, 10 and 50% cumulative volume, respectively.^22,23^

#### Scanning electron microscopy (SEM)

The shape and surface morphology of the particles were studied by the scanning electron microscope (LEO 1430 VP, UK & Germany). Prior to scanning, the samples were coated with a thin layer of gold, using a direct current sputter technique (Emitechk450X, England).

#### In vitro aerosolization assessment 

The *in vitro* aerodynamic parameters including aerodynamic diameter, aerosolization efficiency, dispersion and deposition characteristics of optimized Itraconazole-loaded nanotransfersomal dry powders were assessed by an aerolizer connected to the next generation impactor (NGI) with pre-separator and USP induction port (Copley Scientific, Nottingham, UK).^24^ The NGI was assembled and operated in accordance with USP General Chapter 601 to assess the drug delivered. An appropriate number of hard gelatin capsules (N = 3) were filled with 10 mg of spray dried powder. To ensure efficient particle capture and prevent inter-stage losses due to particle bounce, the particle collection surface of each stage was coated with Tween^®^80. For this purpose, every eight collection cups of the NGI were soaked into Tween^®^80 ethanolic solution (1%) and placed under the fume hood until the complete evaporation of ethanol. The cups were placed into the apertures in the cup tray and the cup tray was located into the bottom frame and lowered into place. The impactor lid was closed with the sealed body attached and the handle was operated to lock the impactor together. Capsules were gathered after actuation for the study of remained powder. The content uniformity test was carried out for 10 mg of each formulation in 5 repetitions. The coefficient variation percentage (CV%) were less than 6% for all spray-dried powders. The batches that showed CV% above 6% were excluded from the study. The induction port was connected to the first stage of the NGI. The flow rate was calibrated using a flow meter (DFM 2000, Copley Scientific, Nottingham, UK) and fixed at 60 L/min. Fine particle fraction (FPF), mass median aerodynamic diameter (MMAD), and geometric standard deviation (GSD) indexes were calculated using the Copley Inhaler Testing Data Analysis Software (CITDAS, version 3.10). The MMAD is defined as the diameter at which 50% of the particles by mass are larger and 50% are smaller. FPF represents the percentage of emitted particles with an MMAD of 5 μm or less estimating the fraction of particles expected to deposit deep within the lungs. The emission was defined as the mass of drug delivered from the inhaler (i.e., total amount excluding the inhaler device and capsule), expressed as a percentage of the total amount of Itraconazole collected. Dispersibility was defined as the ratio of FPF per emission. Each value was expressed as the mean ± standard deviation.^25^

Chloroform as a solvent that can dissolve both SPC and Itraconazole was used as the washing solvent to rupture the transfersomes and extract Itraconazole. The whole process of washing was performed under the fume hood. The capsule shells, pre-separator, USP induction port and NGI stages were washed with suitable amount of chloroform. Chloroform containing extracted drug was moved into the test tubes. The test tubes were kept under nitrogen flow for complete removal of organic solvent. The tubes were filled with certain amount of acetonitrile, vortexed and sonicated for a few minutes to dissolve the extracted Itraconazole completely. 500 µl of acetonitrile was moved into the micro tubes and centrifuged at 13000 rpm for 10 minutes to separate the undissolved transfersome compositions and capsule residuals to yield a particle free solution suitable for HPLC analysis. After deposition onto the stages of the NGI, the mass deposited on each of the impactor pieces was collected and the total mass of drug was quantified by a validated HPLC system.

#### High performance liquid chromatography analysis

The Itraconazole content of each NGI stage was analyzed using a Knauer apparatus HPLC system (Germany) consisted of a model 1000 HPLC pump and a model 2600 tunable absorbance detector, with the following condition: Column: C-18 (150 × 4.6mm, 10 µm, 125 A°) (Germany) protected by a C-18 guard column, mobile phase: acetonitrile: water (90:10), flow rate: 1 mL/min, wave length: 263 nm and injection volume: 20 µl. The run time for Itraconazole appearance following the sample injection was approximately 3 min. The area under the curve (AUC) that demonstrates the Itraconazole concentration was calculated through the machine software (Chromgate^®^ V3.3.1). An excess amount of sample was injected into the injector to make sure that the loop has been completely filled with the sample. Calibration curve was leaner in the range of 1.25 to 25 µg/mL (r^2^=0.997).

#### Statistical analysis

Data are expressed as a mean value ± standard deviation (SD). Statistical analysis was performed using a one-way analysis of variance (ANOVA) with multiple comparisons between deposition data using a Tukey-Kramer HSD test by SPSS software (version 13.0, Chicago, IL, USA). A P value <0.05 was considered statistically signiﬁcant.

## Results

### Preparation of Itraconazole-loaded nanotransfersomes

**Figure 1:**
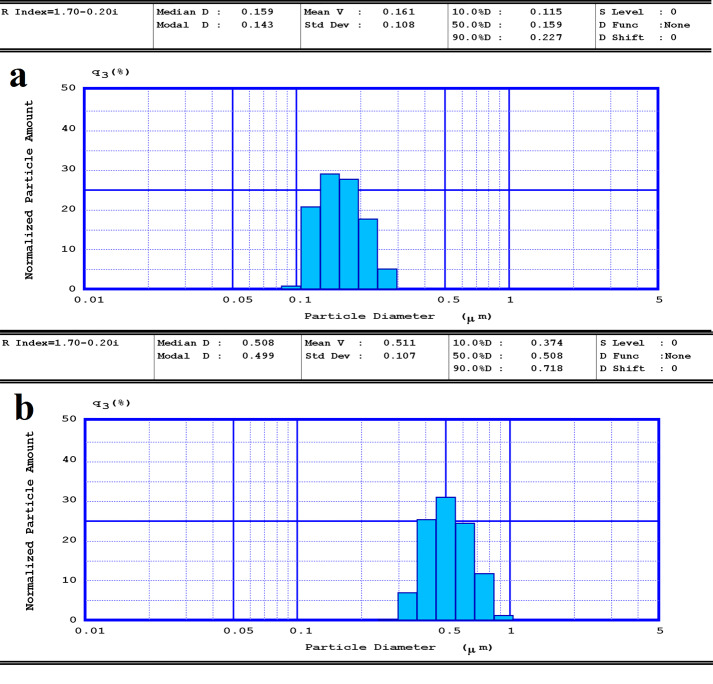
The size distribution pattern of a) drug free nanotransfersomes (T2) and b) drug loaded nanotransfersome (T7) formulations.

The effect of different kinds of surfactants and their concentration on the size of transfersomes are shown in Table 1. The size of blank transfersomes (T1 and T5) were smaller than drug-loaded transfersomes (T3 and T7) with the same compositions. Both drug free and drug-loaded transfersomes showed relatively narrow size distribution pattern (Figure 1). Although there are no statistically difference among the formulations composed of different type and amount of surfactants, formulations containing 10 mg of Span^®^60 (T7) was selected for further experiments due to its lowest size and narrowest size distribution (Table 1). Results also showed that preparation of nanotransfersomal formulations with 10 mg of all types of surfactants resulted in the lowest size compared to using 5 and 15 mg of surfactant.

### Preparation of DPI formulation 

**Figure 2:**
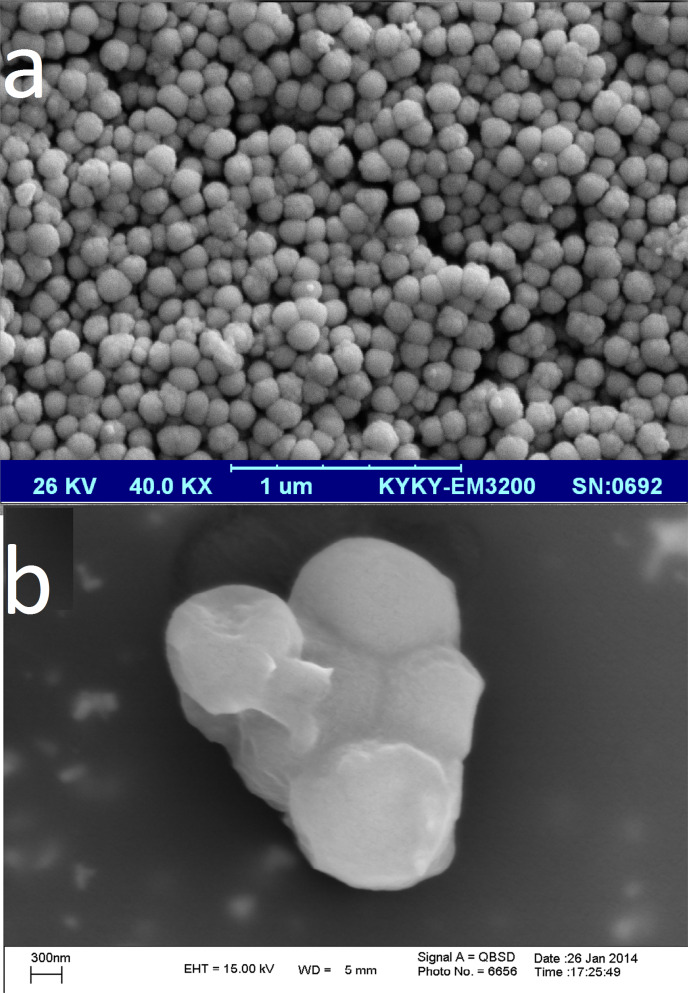
Scanning electron micrographs of a) Itraconazole-loaded nanotransfersomes (formulation T7) and b) those co-spray dried with mannitol (formulation F2).

DPI formulations containing aggregated Itraconazol-loaded nanotransfersomes were prepared by various ratios of transfersomes to mannitol. The formulation F1 was not collected from the cyclone of the spray dryer and mostly adhered to the inner wall of heating chamber. It was reported that lecithin forms a viscous isotropic phase from 88 to 109 °C (the inlet temperature of spray dryer in our experiment) which may be responsible for the formulation adhesion to the spray dryer's chamber. The rest of the formulations with higher amounts of mannitol provided the suitable amounts of powder which were collected from the cyclone (yield value around 50 %) and assessed by NGI for determination of their aerosolization efficacy. Figures 2a and 2b show SEM images of the spray- dried particles (F2), confirmed the formation of aggregated nanoparticles in the suitable range for pulmonary drug delivery.

### In vitro deposition

**Figure 3:**
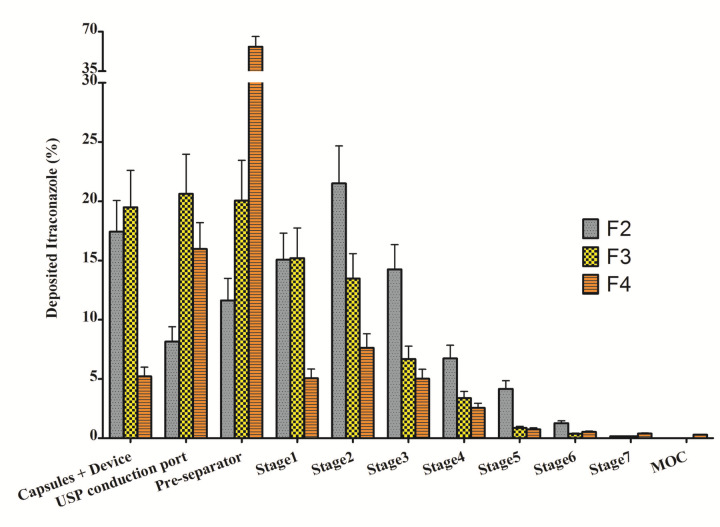
The percentage of Itraconazole from formulations F2, F3 and F4, deposited in various stages of NGI. Data was presented as mean ± standard deviation (n=3).

The amount of Itraconazole in each stage of NGI device was analyzed by HPLC method and illustrated in Figure 3. The in vitro parameters which were calculated according to the results of Figure 3 including FPF, MMAD and GSD are shown in Table 2. F2 showed the highest FPF and dispersibility, therefore, was selected as the optimized formulation. Although F4 had the best emission and GSD indicating its suitable flowability and aerodynamic size distribution, respectively, the main aerosolization indexes (FPF and dispersibility) are lower than other formulations.

**Table 2:** Aerosolization efficiency indexes of selected Itraconazole-loaded nanotransfersomal dry powders measured by the NGI (mean ± SD, n= 3).

**Parameters**	^d^ **F2**	**F3**	**F4**
^a^ **FPF (%)**	37.3 ± 3.1	17.8 ± 2.9	10.4 ± 1.4
^b^ **MMAD (µm)**	5.1 ± 0.7	6.5 ± 1	5.2 ± 0.9
^c^ **GSD**	2.1 ± 0.2	2.1 ± 0.1	1.9 ± 0.1
**Emission (%)**	82.7 ± 2.6	80.5 ± 3.1	94.8 ± 0.8
**Dispersibility**	45.23 ± 2.9	22.11 ± 3.0	10.9 ± 1.1

^a^Fine Particle Fraction, ^b^Mass Median Aerodynamic Diameter, ^c^Geometric Standard Deviation, ^d^F2, F3 and F4 were prepared by co-spray drying of 100 mg nanotransfersome and 200, 300 and 600 mg of mannitol, respectively

## Discussion

Itraconazole is a poorly soluble drug that has displayed a low and variable oral absorption following oral administration. Absolute bioavailability of the oral Itraconazole capsule is 40% lower in the non-fed state compared to the fed state. To obtain a therapeutic level of drug at the site of lung infections, a high oral or intravenous dose must be administered which consequently increases the incidence of unwanted side effects associated with a high drug serum concentration.^26^ In addition, gastrointestinal side effects have been reported in recipients of the oral solution up to 25% in a randomized trial and are the major reason for patient incompliance.^27^ Local targeting of the drug at the usual site of infection (the lungs) means that less total drug may be required for dosing and therefore, the potential for side effects may be minimized due to a much reduced systemic concentration.^28^ It has been reported that pulmonary delivery of nanoparticulate Itraconazole can achieve a significantly higher (more than 10-fold) lung tissue concentrations compared to conventional oral administration of Itraconazole. Effective and sustained lung tissue concentrations were achieved via inhalation of a nanoparticulate Itraconazole formulation.^29^ Additionally, a targeted therapy may also initiate a quicker therapeutic onset and ultimately reduce the duration of treatment.^28^ The utilization of lipidic vesicular systems for pulmonary drug delivery has many potential advantages over aerosol delivery of the corresponding nonencapsulated drug, including universal carrier suitability for most lipophilic drugs, compatibility and preventing local irritation of lung tissue, providing a pulmonary sustained release reservoir prolonging local and systemic therapeutic drug levels and facilitating intracellular drug delivery especially to alveolar macrophages, tumor cells or epithelial cells.^9^ The present study explored a new idea for nanoparticle delivery to the lower respiratory regions of the lung using micrometer-sized carrier particles. In this investigation Itraconazole-loaded nanotransfersomes were incorporated as model drug delivery system by the lipid film hydration technique followed by sonication for size reduction. For the formulation of DPI, prepared nanotransfersomes should have a mean size below 5µm^30^ to form inhalable carrier particles. This requirement was satisfied well when nanotransfersomes were co-spray dried with mannitol, a regularly used inert excipient in pulmonary drug delivery. The administered excipients (lecithin, mannitol, ursodiol, Span^®^60 and Span^®^80) were used because of their biocompatibility and pharmaceutical acceptability. The particle size of the transfersomes with different types of surfactant did not show a significant difference. These results indicate that the particle size of the vesicles was not significantly affected by the type of surfactant.^31^ Spray drying is a low-cost, one-step pharmaceutical process that is widely used in the development of novel dry powder formulations.^3^ Spray drying technique offers a number of potential advantages over lyophilization technique. Spray drying technique was utilized for stabilization of nanoliposomes and development of uniform sized particles with desired aerosolization properties for pulmonary administration to overcome constraints associated with lyophilization technique, such as formation of hard cake, need of micronization, addition of coarse carriers for aersolization, and heterogeneous size distribution pattern.^32^ Once the nanotransfersome aggregates, with their large surface area, in the dry powder formulation is deposited in the aqueous lining fluid in the lung, the mannitol may quickly dissolve, resulting in the dispersion of nanotransfersomes in the respiratory tract fluids.^33^ Carriers form the backbone structure of the solid particles during the spray drying process and facilitate pulmonary delivery.^32^ The carrier of choice for DPI products is currently lactose monohydrate and almost all DPI products already in market are using lactose as a carrier. The advantages of lactose monohydrate are its well-investigated toxicity profile, its broad availability and its relatively low price.^19^ However, it has some disadvantages that should be considered in its usage as carrier for DPIs. Lactose is incompatible with some drugs and compounds with a primary amine group. Lactose may develop a yellow brown color with browning aging that is accelerated by the process of spray drying. Lactose monohydrate is produced from bovine or with bovine-driven additives so that the Transmissible Spongiform Encephalopathy (TSE) discussion is still an issue for this compound. In addition, Lactose intolerance is a situation that requires a solution. For overcoming the above mentioned drawbacks of lactose, mannitol is a suitable substitute carrier that possesses further positive aspects.^20^ For these reasons, mannitol was chosen as an excipient for co-spray drying with nanotransfersomes. Once the nanotransfersomes aggregate, with their large surface area, in the dry powder formulation is deposited in the aqueous lining fluid in the lung, the mannitol may quickly dissolve, resulting in the dispersion of nanotransfersomes in the respiratory tract fluids.^33^ Due to the flexibility in use and high productivity, NGIs (a new impactor type specifically designed for testing pharmaceutical inhalers using the very newest and modern designed theory in 1997) have been used as the most popular testing machine within many inhaler research laboratories. NGI was launched in 2000 and was subsequently accepted into the European Pharmacopeia as Apparatus E and into the United States Pharmacopeia as Apparatus 5 and 6 in 2005. The ideal size for a therapeutic particle is not known exactly but it may be assumed that the MMAD should be not more than 5 µm to pass into the tracheobronchial tree and smaller airways if peripheral deposition is required.^34^ Formulation F2 exhibited an MMAD of 5.1 ± 0.7 µm, and a higher FPF in comparison to the other formulations. In this study, it was shown that increase in mannitol ratio in the spray dried formulations caused a decrease in aerosolization efficiency (Table 2). Maximum FPF of 37.35 ± 3.1% was observed in formulation F2 containing 200 mg of mannitol as compared with F3 (300 mg of mannitol) of 17.8 ± 2.9% and F4 (600 mg of mannitol) of 10.4 ± 1.4%. Previous studies have indicated that increasing the lipid concentration in the spray drying feeds can decrease the residual water content in the powder^35^ which consequently may improve the aerodynamic properties of the DPI formulation. Higher ratios of mannitol improved emission of formulation F4 which may be attributed to the powder flowability improvement by addition of carrier. The findings of this investigation showed that dry powder inhalation formulation of Itraconazole-loaded nanotransfersomes prepared by co-spray drying with mannitol possessed suitable aerosolization performance. Therefore, this formulation may have a potential to deliver Itraconazole to the site of infection in the lungs in the pulmonary aspergillosis. The results highlight the need to examine the enhanced *in vivo* therapeutic effects of pulmonary drug delivery via nanotransfersomal DPI formulations. The results of this study was introduced the potential application of nanotransfersomes in the formulation of DPIs for lung delivery of various drugs.

## Acknowledgments

This paper was extracted from Pharm.D. thesis No. 3662 that was submitted to the Faculty of Pharmacy of Tabriz University of Medical Sciences and financially supported by grant No. 90/92 from the Drug Applied Research Center of the same university.

## Ethical Issues

Not applicable.

## Conflict of Interest

The authors report no conflicts of interest.
